# Guidelines for performing Mendelian randomization investigations: update for summer 2023

**DOI:** 10.12688/wellcomeopenres.15555.3

**Published:** 2023-08-04

**Authors:** Stephen Burgess, George Davey Smith, Neil M. Davies, Frank Dudbridge, Dipender Gill, M. Maria Glymour, Fernando P. Hartwig, Zoltán Kutalik, Michael V. Holmes, Cosetta Minelli, Jean V. Morrison, Wei Pan, Caroline L. Relton, Evropi Theodoratou

**Affiliations:** 1 MRC Biostatistics Unit, University of Cambridge, Cambridge, UK; 2 BHF Cardiovascular Epidemiology Unit, University of Cambridge, Cambridge, UK; 3 MRC Integrative Epidemiology Unit, University of Bristol, Bristol, UK; 4 Population Health Sciences, Bristol Medical School, University of Bristol, Bristol, UK; 5 Division of Psychiatry, University College London, London, UK; 6 Department of Statistical Sciences, University College London, London, WC1E 6BT, UK; 7 K.G. Jebsen Center for Genetic Epidemiology, Department of Public Health and Nursing, NTNU, Norwegian University of Science and Technology, Trondheim, Norway; 8 Department of Health Sciences, University of Leicester, Leicester, UK; 9 Department of Epidemiology and Biostatistics, School of Public Health, Imperial College London, London, UK; 10 Department of Epidemiology and Biostatistics, University of California, San Francisco, San Francisco, CA, USA; 11 Postgraduate Program in Epidemiology, Federal University of Pelotas, Pelotas, Brazil; 12 Swiss Institute of Bioinformatics, Lausanne, Switzerland; 13 Department of Computational Biology, University of Lausanne, Lausanne, Switzerland; 14 University Center for Primary Care and Public Health (Unisanté), Lausanne, Switzerland; 15 MRC Population Health Research Unit, Nuffield Department of Population Health, University of Oxford, Oxford, UK; 16 Clinical Trial Service Unit and Epidemiological Studies Unit, Nuffield Department of Population Health, University of Oxford, Oxford, UK; 17 National Heart and Lung Institute, Imperial College London, London, UK; 18 Department of Biostatistics, University of Michigan, Ann Arbor, MI, USA; 19 Division of Biostatistics, University of Minnesota, Minneapolis, MN, USA; 20 London School of Hygiene & Tropical Medicine, Keppel Street, London, WC1E 7HT, UK; 21 Centre for Global Health, Usher Institute, University of Edinburgh, Edinburgh, UK; 22 Edinburgh Cancer Research Centre, Institute of Genetics and Cancer, The University of Edinburgh, Edinburgh, UK

**Keywords:** Mendelian randomization, guidelines, genetic epidemiology, causal inference

## Abstract

This paper provides guidelines for performing Mendelian randomization investigations. It is aimed at practitioners seeking to undertake analyses and write up their findings, and at journal editors and reviewers seeking to assess Mendelian randomization manuscripts. The guidelines are divided into ten sections: motivation and scope, data sources, choice of genetic variants, variant harmonization, primary analysis, supplementary and sensitivity analyses (one section on robust statistical methods and one on other approaches), extensions and additional analyses, data presentation, and interpretation. These guidelines will be updated based on feedback from the community and advances in the field. Updates will be made periodically as needed, and at least every 24 months.

The aim of this paper is to provide guidelines for performing Mendelian randomization investigations. It is written both for practitioners seeking to undertake analyses and write up their findings, and for journal editors and reviewers seeking to assess Mendelian randomization manuscripts. These guidelines are deliberately written as suggestions and recommendations rather than as prescriptive rules, as we believe that there is no recipe or single “right way” to perform a Mendelian randomization investigation. Best practice will depend on the aim of the investigation and the specific exposure and outcome variables. However, we believe these guidelines will help investigators to consider the key issues in designing, undertaking and presenting Mendelian randomization analyses. These guidelines will be updated based on feedback from the community and advances in the field. Updates will be made periodically as needed, and at least every 24 months.

These guidelines are complementary to the STROBE-MR recommendations on reporting Mendelian randomization investigations ^1, 2^. Here, we provide advice on which analyses to perform in a Mendelian randomization investigation, whereas the STROBE-MR guidelines focus on reporting the analyses chosen by the investigators. We assume a familiarity with the basic concepts of Mendelian randomization and genetic epidemiology, such as pleiotropy and linkage disequilibrium ^3– 6^. We use the term “exposure” to refer to the proposed causal factor, and “outcome” to refer to the trait or disease that the exposure is hypothesized to influence.

Flowcharts highlighting some of the key analytic steps and choices for investigators are provided as Figure 1 and Figure 2, and a one-page checklist summarizing these guidelines written for reviewers of Mendelian randomization analyses is provided as Figure 3. The guidelines are divided into ten sections: motivation and scope, data sources, choice of genetic variants, variant harmonization, primary analysis, supplementary and sensitivity analyses (one section on robust statistical methods and one on other approaches), extensions and additional analyses, data presentation, and interpretation. Software to implement the statistical methods is referenced in Table 1.

**Figure 1.**
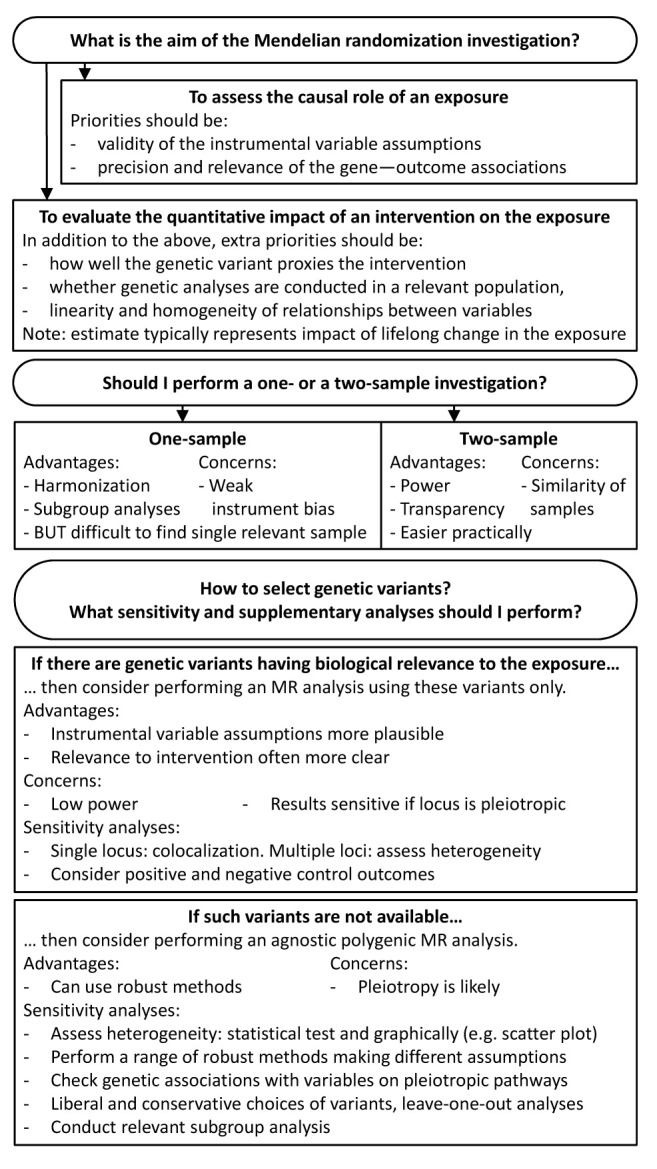
Flowchart highlighting some of the key analytic choices in performing a Mendelian randomization (MR) analysis.

**Figure 2.**
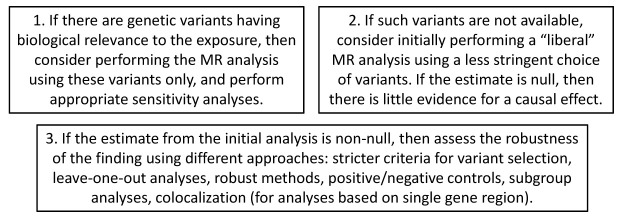
Generic analytic pipeline for Mendelian randomization (MR).

**Figure 3.**
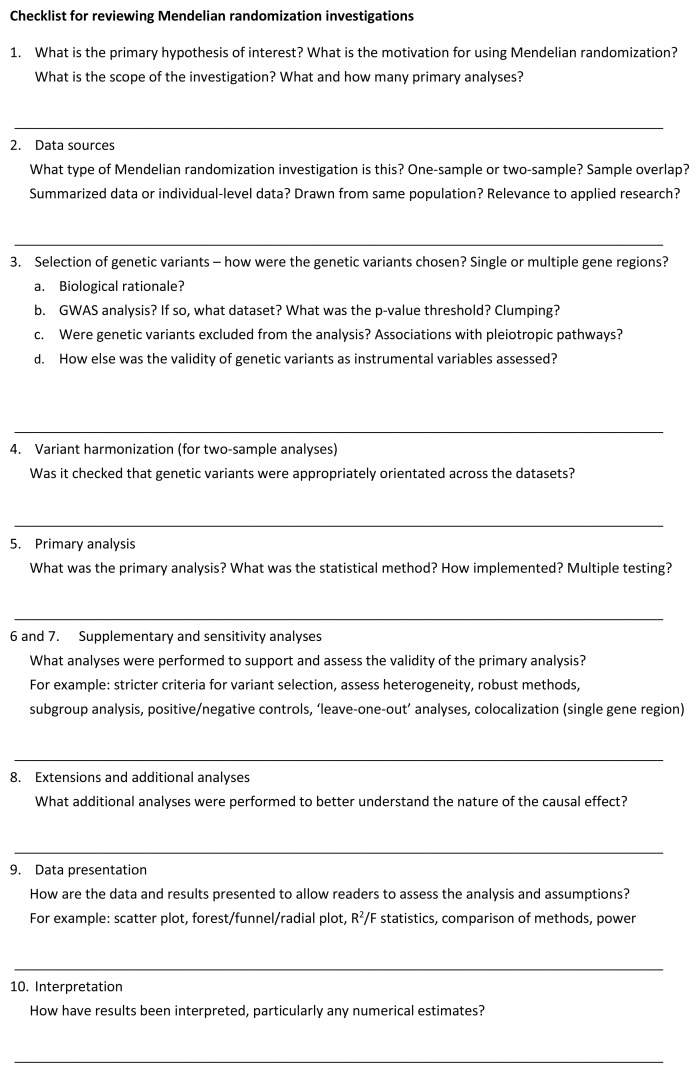
Checklist of questions to consider when reviewing a Mendelian randomization investigation.

**Table 1.** Summary of some methods proposed for Mendelian randomization: inverse-variance weighted method and robust methods.

Method	Consistency assumption	Strengths and weaknesses	Reference	Software
Inverse-variance weighted	All variants valid or balanced pleiotropy	Most efficient (greatest statistical power), biased if average pleiotropic effect differs from zero	16	* †
MR-Egger	InSIDE	Sensitive to outliers, sensitive to violations of InSIDE assumption, InSIDE assumption often not plausible, often less efficient	17	* †
MR-RAPS	InSIDE (except outliers)	Downweights outliers, sensitive to violations of balanced pleiotropy assumption	18	‡
Weighted median	Majority valid	Robust to outliers, sensitive to addition/removal of genetic variants	19	* †
Mode-based estimation	Plurality valid	Robust to outliers, sensitive to bandwidth parameter and addition/removal of genetic variants, generally conservative	20	* †
MR-PRESSO	Outlier-robust	Removes outliers, efficient with valid IVs, very high false positive rate with several invalid IVs	21	‡
MR-Robust	Outlier-robust	Downweights outliers, efficient with valid IVs, high false positive rate with several invalid IVs	22	*
MR-Lasso	Outlier-robust	Removes outliers, efficient with valid IVs, high false positive rate with several invalid IVs	22	
Contamination Mixture	Plurality valid	Robust to outliers, sensitive to variance parameter and addition/ removal of genetic variants	23	*
MR-Mix	Plurality valid	Robust to outliers, requires large numbers of genetic variants, very high false positive rate in several scenarios	24	‡
MR-cML	Plurality valid	Likelihood-based and robust to the violation of all three IV assumptions	25	* ‡

Each of the methods in the table can be implemented using summarized data. False positive rates refer to the simulation study by Slob and Burgess ^26^. InSIDE is the Instrument Strength Independent of Direct Effect assumption. IV = instrumental variable. * Implemented in MendelianRandomization package for R ( https://cran.r-project.org/web/packages/MendelianRandomization/index.html) † Implemented in mrrobust package for Stata ( https://github.com/remlapmot/mrrobust) ‡ Implemented for R in its own software package: - MR-PRESSO in mrpresso package ( https://github.com/rondolab/MR-PRESSO), - MR-RAPS in mr.raps package ( https://github.com/qingyuanzhao/mr.raps), - MR-Mix in MRMix package ( https://github.com/gqi/MRMix), - MR-cML in MRcML package ( https://github.com/xue-hr/MRcML).

## 1. Motivation and scope

Mendelian randomization uses genetic variants to assess causal relationships using observational data. A genetic variant can be considered as an instrumental variable for a given exposure if it satisfies the instrumental variable assumptions: 1) it is associated with the exposure, 2) it is not associated with the outcome due to confounding pathways, and 3) it does not affect the outcome except potentially via the exposure ^7, 8^.

Before embarking on a Mendelian randomization analysis, investigators should consider the aims of their investigation and the primary hypotheses of interest. There are many potential motivations for using Mendelian randomization, and the motivation should influence decisions on how to perform the analysis, and how to arrange and present its results. The objective of a Mendelian randomization analysis is a test of a causal hypothesis, and sometimes additionally an estimate of a causal effect ^9^. The straightforward statement of the causal hypothesis is that interventions on the exposure variable will affect the outcome. If the genetic associations with the exposure vary with time, then there are some nuances in terms of what causal hypotheses can be tested ^10^; we discuss the impact of time-varying relationships between variables in Section 10.

If a Mendelian randomization investigation is performed primarily to assess whether an exposure has a causal effect on an outcome, then estimating the size of the causal effect of the exposure on the outcome is less important and may even be unnecessary ^9, 11^. Priorities in such an analysis are to find genetic variants that satisfy the instrumental variable assumptions and to test their associations with the outcome in the largest available dataset that is relevant to the causal question of interest. Investigators may be able to find mediating traits downstream of the exposure that both help understand the mechanistic pathways from the exposure to the outcome, and provide modifiable targets for intervention in order to influence the outcome.

In contrast, if investigators seek to estimate the quantitative impact on the outcome of a proposed intervention in the exposure ^12^, then further questions become more important, such as how well the genetic variant proxies the specific intervention, whether genetic associations with the exposure are estimated in a relevant population, and whether the relationships between variables are linear and homogeneous in the population ^13^. However, as we discuss in Section 10, causal estimates from Mendelian randomization should always be interpreted with caution. Alternatively, if investigators simply want to assess whether traits share common genetic predictors (potentially implying shared aetiological mechanisms), then an analytic approach that assesses shared heritability (such as LD-score regression ^14^ or bivariate genome-based restricted maximum likelihood [GREML] ^15^) may be preferable to conducting a Mendelian randomization investigation.

Investigators should also give thought to the scope of their analysis. If the aim of the investigation is to understand disease aetiology, then consideration of a limited set of exposures/outcomes as main analyses may be justified. Whereas if the question relates to public health, then consideration of a wider range of outcomes influenced by an exposure may be worthwhile, as public health recommendations should assess the broad consequences of intervention on an exposure, which may involve weighing risks and benefits for different outcomes. At the extreme end of the spectrum is a phenome-wide Mendelian randomization investigation, in which very large numbers of exposure/outcome pairs are considered ^27– 29^. Such analyses are generally regarded as exploratory or “hypothesis-generating”, and results are typically treated as provisional until replicated in an independent dataset.

Specifying the primary analyses in a Mendelian randomization investigation is important to address problems of multiple testing, particularly given the large number of analyses that could be performed using available genetic data ^30^. Additional analyses, including subgroup analyses and analyses on related outcomes may be presented as supplementary, exploratory, or sensitivity analyses. An overly conservative approach to multiple testing is often excessive, given the typically low power of Mendelian randomization studies and the fact that Mendelian randomization often investigates exposure/outcome relationships with prior epidemiological or biological support. As with all epidemiological analyses, selective reporting of “significant” results (leading to reporting bias) should be avoided and all analyses performed should be described transparently.

## 2. Data sources

The next fundamental question is which data sources will be used: how many datasets are included in the analysis and whether the analysis is performed using individual-level data or summarized data.

Mendelian randomization investigations can be performed using data from a single sample (known as one-sample Mendelian randomization), in which genetic variants, exposure, and outcome are measured in the same individuals, or from two samples (known as two-sample Mendelian randomization), in which variant—exposure associations are estimated in one dataset, and variant—outcome associations are estimated in a second dataset ^31^. Two-sample investigations often occur when genetic associations with the exposure are estimated in a cross-sectional sample of healthy individuals, to reflect genetic associations with usual levels of the exposure in the population, and genetic associations with a binary disease outcome are estimated in a case-control study.

There are benefits and limitations of both one- and two-sample settings. A one-sample setting allows the investigation to be conducted in a single population sample, meaning that Mendelian randomization and conventional epidemiological findings (for example from multivariable-adjusted regression) can be compared in the same individuals. In a two-sample setting, the populations from which the two samples were extracted may differ. This is problematic if associations of the genetic variant with the exposure or with variables on pleiotropic pathways differ between the two samples, as this could affect the validity of the instrumental variable assumptions. A particular concern arises if the two samples represent different ethnic groups, as patterns of linkage disequilibrium can differ between population ancestry groups, meaning that a genetic variant may not be as strongly (or even not at all) associated with the exposure in the outcome dataset. Alternatively, the two samples could differ substantially according to population characteristics such as age, sex, socio-economic background, and so on ^32^. Such differences can affect not only the interpretation of causal estimates, but also the validity of causal inferences ^33^. For example, genetic variants associated with smoking intensity may be strongly associated with disease outcomes in populations where smoking is common, but not in populations where smoking is rare. One-sample analyses do not suffer from these concerns, nor do they require harmonization of the genetic variants across the datasets (see Section 4).

Another related issue is whether the analysis is performed using individual-level data or summarized data. Summarized data are genetic association estimates from regression of the exposure or outcome on a genetic variant ^16, 34^. Several large consortia have made such estimates publicly available for millions of variants ^30, 35, 36^. Although the use of summarized data is often synonymous with the two-sample setting, the benefits and limitations for the analysis of the two choices (i.e. one- versus two-sample and individual-level versus summarized data) are distinct. Moreover, two-sample approaches can be used with individual-level data (such as the use of externally-derived weights), and summarized data approaches can be used with one-sample data (if necessary, by creating the summarized data from the individual-level data ^37^).

Summarized data are often available for larger sample sizes, meaning that power to detect a causal effect is increased. However, access to only summarized data limits the range of analyses that can be performed. Individual-level data are required to conduct analyses in specific subgroups or strata of the population, or to choose which variables to adjust for when generating the summarized data. If published summarized association estimates have already been adjusted for a variable causally downstream of the exposure or outcome, collider bias (see Section 7) can occur ^38^. Individual data in a one-sample setting are required to investigate non-linear effects ^39^. A specific advantage of publicly available summarized data is transparency, as the analysis can be reproduced by a third party with access to the same data.

One- and two-sample investigations also differ in terms of bias with weak instruments ^40^. In a one-sample setting, if the genetic variant–exposure associations are weak, then chance variation means that genetic associations with the exposure and outcome are correlated in the direction of the confounded association between the two. This results in instrumental variable estimates that are biased in the direction of the confounded association, and inflated false positive (type 1 error) rates, particularly when more than one variant is included in the analysis ^41^. In a two-sample setting without sample overlap, bias due to weak instruments is in the direction of the null, and does not lead to false positive findings. However, as several large consortia have overlapping studies, participants may overlap between the datasets used to estimate the genetic associations with the exposure and outcome ^42^. In this case, the direction and size of the bias varies linearly depending on the degree of overlap (formally, depending on the degree of correlation between the genetic association estimates). For the special case of a one-sample analysis with a binary disease outcome, if the genetic associations with the exposure are estimated in the controls only, then genetic associations with the exposure and outcome will not be correlated, and bias will follow the pattern of the two-sample setting ^42^. Various statistical methods have been proposed to reduce weak instrument bias due to sample overlap and bias due to winner’s curse (see Section 3) ^43– 45^.

The “randomization” in Mendelian randomization refers to the quasi-random allocation of genetic variants from parents to offspring that occurs at conception. This randomization only truly holds conditional on the parental genotype. The key consequence of this randomization is that genetic variants are independently distributed from traits that they do not affect, an implication of Mendel’s laws of segregation and independent assortment. There is some plausibility that this independence holds for many traits at a population level in large “well-mixed” populations. Empirical investigations in European populations have shown that associations between genetic variants and many traits are no stronger than would be expected due to chance alone ^46, 47^.

Concerns about independence are greater for traits that are more socially-patterned, as this increases susceptibility to associations arising from population structure, assortative mating, and dynastic effects ^48^. Population structure can give rise to genetic associations due to differences in the frequency of a variant and the distribution of a trait across the population (such as latitude in Europe, which correlates with allele frequencies for lactase persistence variants and milk consumption ^49^). Assortative mating occurs when individuals reproduce with people who are more similar to themselves than would be expected by chance. This can also lead to genetic associations that represent social differences rather than causal effects ^50^. Finally, dynastic effects occur when a parent’s genotype affects their child’s outcome by a causal pathway not acting via the child’s phenotype (for example, due to an effect of the parent’s phenotype on the child’s outcome). This can induce associations between the offspring’s genotype and outcomes that do not reflect the effect of the exposure in the offspring.

These potential sources of bias have encouraged the development of statistical approaches and datasets to perform within-family Mendelian randomization analyses, exploiting the random allocation of variants between siblings ^51^. Hence, if a relevant dataset is available and statistical power is reasonable, investigators could consider performing a within-family Mendelian randomization analysis, particularly if the exposure is socially-patterned or likely to be subject to population stratification ^48, 52^. If this is not possible, then the validity of the investigation relies on independence of the genetic variants from potential confounders holding at a population level. However, within-family analyses typically have lower power than analyses in unrelated individuals, as families where all individuals have the same genotype (such as where both parents are major homozygotes) would not contribute any information to the analysis. Hence, imprecise null findings from family-based analyses should be interpreted with caution, particularly if a population-based analysis suggests a causal effect.

## 3. Selection of genetic variants

The most important decision to be made in designing a Mendelian randomization investigation is which genetic variants to include in the analysis ^53^. First, it is necessary to decide whether the analysis is performed using variants from a single gene region, or using variants from multiple regions of the genome (a polygenic analysis). For example, a Mendelian randomization analysis for C-reactive protein (CRP) may be conducted using variants in the neighbourhood of the *CRP* gene region (which encodes C-reactive protein), or it may be conducted using all independent genome-wide significant predictors of CRP ^54^. The former has advantages of specificity – if a gene region has a specific biological link with the exposure, then the Mendelian randomization investigation based on these variants (sometimes called a “ *cis*-Mendelian randomization analysis” ^55^, as the variants are *cis*-variants for the gene product) is more plausible as an assessment of the causal role of that particular exposure compared with an analysis including all genome-wide significant predictors of CRP regardless of function. However, if only one gene region is included in the analysis, then several robust statistical analysis methods (see Section 6) are less reliable, as they assume independence in whether variants violate the instrumental variable assumptions. Variants in the same gene region are likely to either all be valid instruments or all invalid. When genetic variants are all valid instruments, power depends on the proportion of variance in the exposure explained by the variants ^56^ – hence a polygenic Mendelian randomization investigation will typically have greater power than one including variants only from a single gene region.

Mendelian randomization analyses for investigating drug targets often use variants in a single gene region, typically the gene that encodes the protein target under investigation ^57, 58^. For example, investigations into the effects of glucagon-like peptide 1 receptor (GLP1R) agonists have considered variants in the *GLP1R* gene region ^59^, and investigations into the effects of activated factor X inhibitors have considered variants in the *F10* gene ^60^. However, for complex multifactorial exposures such as body mass index or blood pressure, there is no single relevant gene, and so a more agnostic polygenic analysis may be necessary. In some cases, both approaches may be possible: for example, variants associated with low-density lipoprotein (LDL) cholesterol in the HMGCR gene region have particular relevance for understanding the impact of taking statin drugs, whereas a polygenic analysis including genetic predictors of LDL-cholesterol from multiple gene regions may be informative about the effect of LDL-cholesterol perturbation more generally. The latter approach allows investigators to test for consistency of the causal finding across multiple variants that influence the exposure via different biological pathways ^61^.

When the analysis is based on a single gene region, it may be that a single variant is included in the analysis. However, if multiple variants explain independent variance in the exposure, then their inclusion will increase the power to detect a causal effect, even if the variants are partially correlated. With summarized data, appropriate methods should be used to account for correlated variants ^32^. For drug targets, variants may be chosen based on associations with levels of a protein or similar biomarker that reflects pharmacological perturbation of the target, or expression of the targeted gene in a relevant tissue or cell type. If there are many correlated candidate variants in a gene region, then including all variants in a single analysis will typically result in numerical instability, as the analysis can be highly sensitive to small changes in the variant correlation matrix ^62^. Variable selection and dimension reduction approaches have been proposed to maximize the proportion of variance in the exposure explained by the selected variants while avoiding instability due to multicollinearity ^63^.

For a polygenic analysis, there are two main strategies for selecting variants: either a biologically driven approach or a statistically driven approach. The two approaches are not mutually exclusive, and the overall decision of which variants to include may comprise elements from both approaches.

A biological approach to selecting genetic variants would include variants from regions that have a biological link to the exposure of interest. For example, several Mendelian randomization investigations for vitamin D have used variants from four gene regions that are biologically implicated in the synthesis or metabolism of vitamin D ^64^. However, caution is required as biological understanding is often imperfect. As an example, although genetic variants in the *IL6R* gene region are associated with increased circulating levels of interleukin-6, they in fact decrease interleukin-6 signalling, leading to opposite directions of association with disease outcomes to those expected based on serum interleukin-6 measurements ^65^.

A common statistical approach when selecting genetic variants is to include all variants associated with the exposure of interest at a given level of statistical significance (typically, a genome-wide significance threshold, such as p < 5×10 ^-8^). Selection may be based on the dataset in which genetic associations with the exposure are estimated. However, this can lead to “winner’s curse” –genetic associations tend to be overestimated in the dataset in which they were first discovered. If genetic variants are selected based on their associations with the exposure in the dataset under analysis, weak instrument bias is exacerbated (in the direction of the observational association in a one-sample setting, and in the direction of the null in a two-sample setting) ^41^. This bias can be avoided by selecting genetic variants based on a different dataset entirely. This can lead to a “three-sample” analysis, in which variants are identified in one dataset, and the genetic associations with the exposure and outcome are estimated in separate datasets ^66^. If associations with the exposure from separate large datasets are not available, investigators will have to choose between basing their variant choice on the dataset under analysis (and hence risking winner’s curse bias), or basing their variant choice on a smaller dataset (and hence risking uninformative findings due to low power) ^67^. When genetic variants are chosen solely based on their association with the exposure without reference to the function of the variants, researchers should be especially careful about the possibility of variants being pleiotropic.

A more nuanced approach to variant selection would be to start off with a statistical rationale for choosing genetic variants, but then to exclude variants that are known to be pleiotropic or that are associated with variables that represent pleiotropic pathways to the outcome. However, a genetic association with a variable does not necessarily reflect that the instrumental variable assumptions are violated. Additionally, if variants are associated with a variable that has no influence on the outcome, bias will not be introduced.

**Figure 4.**
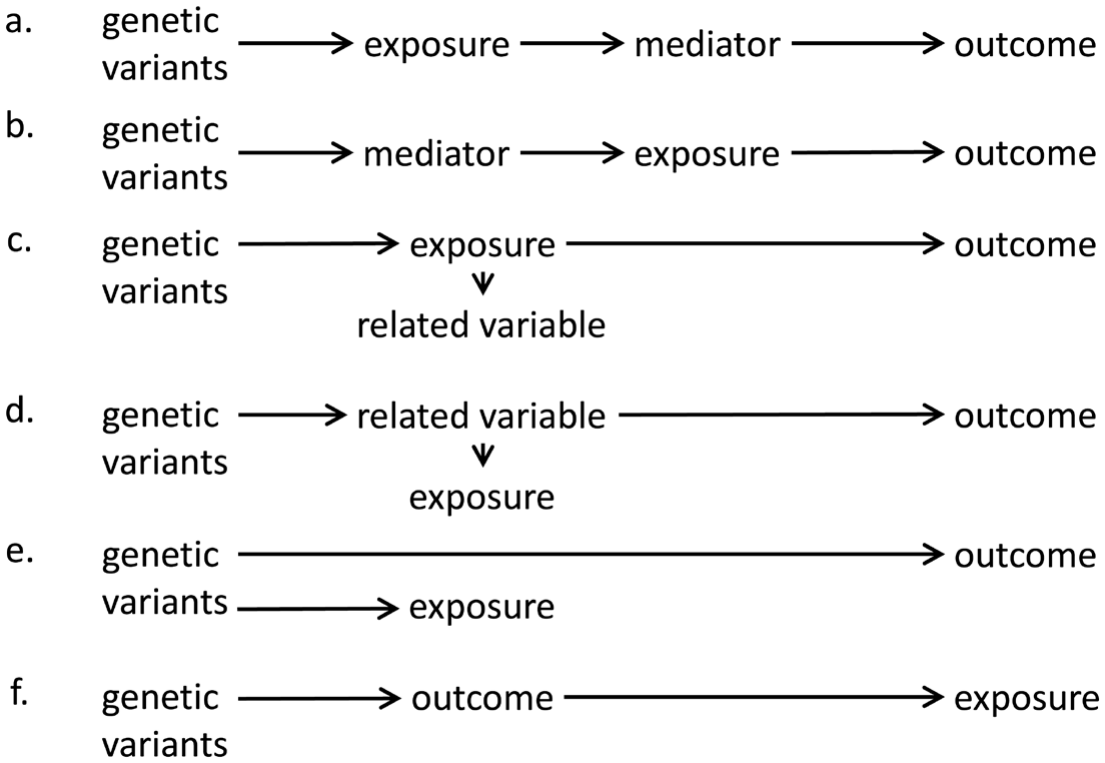
Directed acyclic graphs illustrating validity and invalidity of instrumental variable assumptions in different scenarios. **a**) Mediator is on causal pathway from exposure to outcome. **b**) Mediator is on causal pathway from genetic variants to exposure. **c**) Genetic variants influence the exposure, which has downstream effect on a related variable which does not affect the outcome. **d**) Genetic variants influence a related variable, and the related variable affects the outcome and exposure of interest. We note that the related variable may be known or unknown. **e**) Genetic variants influence the exposure and outcome via different causal pathways. **f**) Genetic variants influence the outcome primarily, and only influence the exposure via the outcome. In scenarios **a**, **b**, and **c**, as there is no alternative pathway from the genetic variants to the outcome, the instrumental variable assumptions are satisfied. In scenario **d**, the pathway from the genetic variants to the outcome does not pass via the exposure, and so the instrumental variable assumptions are not satisfied for the exposure (although they are satisfied for the related variable). Scenarios **a**, **b**, and **c** are examples of “vertical pleiotropy” (also called “indirect pleiotropy”) that do not invalidate the instrumental variable assumptions. Scenario **d** reflects a situation where the causal risk factor has been incorrectly identified – it is not the exposure, but the related variable. Scenario **e** reflects “horizontal pleiotropy” (also called “direct pleiotropy”) that violates the instrumental variable assumptions. Scenario **f** reflects a reverse causation situation where the genetic variant has been incorrectly identified as primarily affecting the exposure.

We use the term “horizontal pleiotropy” (sometimes referred to as “direct pleiotropy” or simply “pleiotropy”) to refer to the scenario where a genetic variant is associated with variables on different causal pathways to the outcome, and “vertical pleiotropy” (sometimes referred to as “indirect pleiotropy” or “mediated pleiotropy”) to refer to the scenario where a genetic variant is associated with variables that are on the same causal pathway to the outcome ^68^. Provided that the causal pathway from the genetic variant to the outcome is mediated entirely via the exposure (see Figure 4), a genetic variant is a valid instrument for assessing the causal role of the exposure (assuming the other instrumental variable assumptions are satisfied), even if it is associated with another variable ^31^. In practice, distinguishing between horizontal pleiotropy and vertical pleiotropy requires knowledge of the relationships between the variables in the analysis. When there are multiple genetic variants, horizontal pleiotropy is more likely if a genetic association with a specific variable is only observed for a small number of variants. In contrast, vertical pleiotropy (in particular corresponding to the scenarios in Figure 4a–c) is likely to lead to genetic associations with that variable for all variants that associate with the exposure. While removing horizontally pleiotropic variants from a Mendelian randomization analysis should lead to more reliable results, care must be exercised, as removing vertically pleiotropic variants could lead to distorted causal estimates.

Another possible scenario that would lead to instrument invalidity is if genetic variants influence the outcome primarily rather than the exposure ( Figure 4f, see also discussion on reverse causation in Section 7). If there is a reverse causal effect of the outcome on the exposure, then genetic predictors of the outcome could be identified as hits in a genome-wide association study for the exposure. However, such variants would not be valid instrumental variables.

In conclusion, there is no one correct way to choose which genetic variants to include in an analysis. Causal conclusions will be more reliable when the instrumental variable assumptions are more plausible. In practice, a balance may need to be struck between including fewer variants (and potentially having insufficient power) and including more variants (and potentially including more pleiotropic variants). We note the possibility that a researcher could exploit this uncertainty and perform a data-driven investigation, choosing variants based on the results of Mendelian randomization analyses with different sets of variants. This underscores the importance of writing an analysis plan before looking at the data, and considering prospectively what criteria may be considered for including and excluding genetic variants from the analysis. This problem is not unique to Mendelian randomization, and pre-registration of analysis plans has been suggested as a potential way of ensuring that analyses are conducted transparently and without bias (whether intentional or unintentional) ^69^.

A practical suggestion for performing a polygenic analysis is to consider both a liberal analysis, including more genetic variants, and a conservative analysis, including fewer variants ^31^. While it is theoretically possible for pleiotropy to lead to a false negative finding, it is generally more likely that pleiotropy will bias estimates away from the null. Hence a null finding in a liberal analysis is more convincing evidence of a true null relationship – there is little evidence for a causal relationship even when potentially pleiotropic genetic variants are included in the analysis. Section 6 and Section 7 describe sensitivity analyses for assessing the instrumental variable assumptions and the robustness of non-null findings.

## 4. Variant harmonization

Genetic associations with exposures and outcomes are typically reported per additional copy of a particular allele. Hence, when combining summarized data on genetic associations, it is important to ensure that genetic associations are expressed per additional copy of the same allele ^70^. This is particularly important as not all publicly-available data resources are consistent about reporting strand information correctly. For example, if a genetic variant is a biallelic single nucleotide polymorphism (SNP) with alleles A and G on the positive strand, then the corresponding base pairs on the negative strand will be T and C. In this case, one dataset may report the association per additional copy of the A allele, and another per additional copy of the T allele – but the same comparison is being made. Allele and strand information can be double-checked by comparing allele frequency information – if the allele frequencies are similar for the A and T alleles, then the researcher can be more confident that this is a strand mismatch. Additional care should be taken for palindromic variants – if the alleles were A and T (or C and G), then the same alleles would appear on both the positive and negative strands. In such a case, if the allele frequency is close to 50%, analysts may choose to drop the variant from the analysis if it is not possible to verify that the alleles have been correctly orientated. While this is a conservative policy, allele alignment problems have led to incorrect results in Mendelian randomization analyses, and retractions and corrections of manuscripts.

## 5. Primary analysis

Different statistical methods have been proposed for Mendelian randomization with individual-level data and with summarized data. In a one-sample setting with individual-level data, a causal effect estimate can be obtained using the two-stage least-squares (2SLS) method. In the first stage, the exposure is regressed on the genetic variants and any relevant covariates. In the second stage the outcome is then regressed on the predicted values of the exposure from the first regression and the same covariates ^71^. In general, we recommend only including as covariates age, sex, genomic principal components of ancestry, and technical covariates (such as recruitment centre), as further adjustment may bias estimates either if adjustment is for a variable on the causal pathway from the genetic variants to the outcome (a mediator), or if adjustment induces collider bias ^72^. Strictly speaking, the 2SLS method refers to a two-stage analysis using linear regression for continuous outcomes and exposures. Similar two-stage analyses can be performed with binary variables using logistic regression ^73^, although in this case estimates are sensitive to correct specification of the first-stage regression model ^74^ and other approaches that make weaker distributional assumptions, such as structural mean models, may be preferred ^75^.

The 2SLS method can be applied to the two-sample setting if individual-level data are available for both samples ^76^. However, it is typical for two-sample investigations to use summarized data. With summarized data, if only one genetic variant is used as an instrument, the causal effect estimate is simply the ratio of the variant—outcome association divided by the variant—exposure association. With multiple variants as instruments, the most commonly used method is the inverse-variance weighted (IVW) method ^16^. With uncorrelated variants, the IVW estimate can be obtained from an IVW meta-analysis of the ratio estimates for the individual variants ^77^. The same estimate can equivalently be calculated as the ratio estimate using a weighted genetic risk score as a single instrument, with the weights equal to the associations of each variant with the exposure estimated in the first sample ^32^. A modification of this method has been proposed to allow for correlation (linkage disequilibrium) between variants ^32^. For continuous outcomes, the IVW estimate is asymptotically equivalent to the 2SLS estimate obtained from individual level data ^16^. The 2SLS method (and thus also the IVW method) is the most efficient estimate of the causal effect when all genetic variants are valid instruments ^32^.

If all genetic variants are valid instruments and the relationships between all variables (genetic variants, exposure and outcome) are linear and homogeneous for all individuals in the population, then we would expect the variant-specific estimates (that is, the ratio estimates based on each variant in turn) to all target the same causal parameter, and for there to be no more heterogeneity between the variant-specific estimates than would be expected by chance alone ^13^. However, there are many reasons why excess heterogeneity may occur in practice. These include statistical reasons (such as departures from linearity and homogeneity across individuals) and biological reasons. For instance, variants associated with body mass index (BMI) influence BMI via different biological mechanisms ^78^. Additionally, some variants are associated with BMI from early childhood and others from adolescence or later. Variants that influence BMI for longer may be expected to have stronger proportional associations with chronic disease outcomes for which BMI is a cause. Hence if there is a true causal effect of the exposure on the outcome, some heterogeneity may be expected in the variant-specific causal estimates. However, heterogeneity would also arise if some genetic variants are not valid instrumental variables (see Section 6) ^79^.

The IVW method can be performed using a fixed-effects or a random-effects meta-analysis model. Unless there are very few variants (meaning that heterogeneity between the variant-specific estimates cannot be estimated reliably) or all variants are taken from the same gene region, we recommend using a multiplicative random-effects model as the default option for the IVW method. If there is no more heterogeneity between the ratio estimates for the individual variants than would be expected by chance alone, then the random-effect analysis is equivalent to the fixed-effect analysis, and there is no loss of precision in making the weaker random-effects assumption. However, if there is excess heterogeneity, then the fixed-effect analysis is inappropriate, as its confidence intervals are misleadingly narrow. A multiplicative random-effects model is preferred to the additive random-effects model that is more common in the meta-analysis literature as it does not change the relative weighting of the variant-specific estimates ^34^. In contrast, an additive random-effects model upweights outlying estimates, which are more likely to represent pleiotropic variants. The multiplicative random-effects IVW method provides valid causal estimates under the assumption of balanced pleiotropy; that is, pleiotropic effects on the outcome are equally likely to be positive as negative ^34^.

We recommend the IVW method with multiplicative random-effects as the primary analysis method for use with summarized data, because it is the most efficient analysis method with valid instrumental variables, and it accounts for heterogeneity in the variant-specific causal estimates. If a causal effect is detected using this method, then investigators should proceed to perform sensitivity analyses ( Section 6 and Section 7) to assess the robustness of their finding to the assumption of balanced pleiotropy.

A scenario that requires a different approach to the primary analysis occurs when there are several related exposures that have shared genetic predictors, meaning that it is difficult to find specific predictors of the individual exposures. In this case, a multivariable Mendelian randomization approach may be the primary analysis strategy ^80^. Multivariable Mendelian randomization is an extension to standard (univariable) Mendelian randomization that allows genetic variants to be associated with more than one exposure, and estimates the direct causal effects of each exposure in a single analysis model. The instrumental variable assumptions in multivariable Mendelian randomization require each variant to be associated with at least one of the exposures, not associated with the outcome via confounding, and not to affect the outcome except potentially via its association with one or more of the exposures included in the analysis model. For identification, it is also required that there is no perfect collinearity between the genetic associations; that is, there are variants that explain independent variation in each exposure ^81^. Examples of exposure sets where multivariable Mendelian randomization has been used include lipid fractions (such as high-density lipoprotein cholesterol, LDL-cholesterol, and triglycerides) ^82^, and body composition measures (such as fat mass and fat-free mass) ^83^. Provided that genetic variants act as instrumental variables for the set of exposures, the direct causal effects of the individual exposures on the outcome can be estimated ^84^. Both the 2SLS and IVW methods can be adapted to the multivariable setting ^81^. A multivariable analysis strategy may also be worthwhile if genetic variants are associated with measured exposures that represent potentially pleiotropic pathways from the genetic variants to the outcome, as the effects of these exposures on the outcome will be accounted for in the multivariable analysis model ( Section 7). Specific methods have been proposed based on a multivariable approach in the context of gene expression data, such as the transcriptome-wide summary statistics-based Mendelian randomization (TWMR) method ^85^.

## 6. Robust methods for sensitivity analysis

A robust analysis method is defined here as a method that can provide valid causal inferences under weaker assumptions than the standard IVW method. Many robust analysis methods are available to detect and account for pleiotropy when using multiple genetic variants. Any polygenic Mendelian randomization investigation where variants are chosen based on their associations with the exposure that does not perform one or more robust methods may be viewed as somewhat incomplete ^54, 86^. Investigators should consider using multiple methods that make different assumptions about the nature of the underlying pleiotropy ^26^. Although robust methods typically use the term ‘pleiotropy’, any source of instrument invalidity can be expressed as algebraically equivalent to bias from pleiotropy ^87^, and so these methods can help assess sensitivity of findings to instrument invalidity more generally, and not simply invalidity that arises from horizontal pleiotropy. However, the robust methods are more likely to be effective for addressing instrument invalidity that arises due to issues such as pleiotropy or linkage disequilibrium with a variant influencing a confounder, which affect specific variants in a sporadic way, and less effective for instrument invalidity that arises due to issues such as population stratification or dynastic effects, which affect all variants in a systematic way ^48^. We here use the language of pleiotropy to make mathematically precise statements about the assumptions needed for methods to provide consistent estimates, but these statements cover instrument invalidity more generally.

While a full comparison of all the robust methods that have been proposed is beyond the scope of this paper, a summary of several methods is provided as Table 1. This table is based on a broader review and comparison of methods ^26^. We proceed to provide a brief description of some commonly used methods.

The most commonly used robust methods are MR-Egger, median- and mode-based methods, and MR-PRESSO. We focus on these methods here as they can be implemented using summarized data alone, and they rely on different assumptions to provide consistent causal estimates. The MR-Egger method estimates the causal effect as the slope from the weighted regression of the variant—outcome associations on the variant—exposure associations, and the average pleiotropic effect as the intercept. The method allows all genetic variants to have pleiotropic effects; however, it requires that the pleiotropic effects are independent of the variant–exposure associations (referred to as the Instrument Strength Independent of Direct Effect (InSIDE) assumption) ^17^. This assumption would be violated in the case of “correlated pleiotropy”, which occurs when genetic variants influence a confounder of the exposure and outcome (and hence there are correlated pleiotropic effects on the exposure and outcome) ^88^. A multivariable version of the MR-Egger method is available ^89^. Estimates from the MR-Egger method are particularly affected by outlying and influential datapoints ^90^, and are prone to be imprecise, particularly when the variant—exposure associations are all similar in magnitude. This can lead to the method having low power to detect a causal effect. A heterogeneity measure has been proposed to quantify the similarity between variant—exposure associations and the potential impact on MR-Egger analyses ^91^. Another method making the InSIDE assumption is the MR-RAPS (robust adjusted profile score) method, which first excludes strongly pleiotropic variants, and then assumes all remaining variants follow the InSIDE assumption ^18^.

The median- and mode-based methods ^19, 20, 92^ rely on some genetic variants being valid instruments, but make weaker assumptions about the invalid instruments and are more robust to outliers. Specifically, the median-based method assumes that less than half of the variants are invalid instruments (majority valid assumption), and the mode-based method assumes more variants estimate the true causal effect than estimate any other quantity (plurality valid assumption). Intuitively speaking, both methods take the variant-specific causal estimates (i.e. the ratio estimates based on the individual variants), and calculate a measure of central tendency of these estimates. These methods have a natural robustness to variants with outlying ratio estimates, and so are not as affected by the presence of a small number of pleiotropic variants as the IVW and MR-Egger methods. The mode-based method has been shown to have low precision in some simulated and real datasets ^26^. Other methods have been proposed that make the same plurality valid assumption as the mode-based method, including the contamination mixture method ^23^ and MR-Mix ^24^.

The MR-PRESSO method is a variation on the IVW method that first sequentially removes genetic variants from the analysis whose variant-specific causal estimate differs substantially from those of other variants ^21^. The IVW method is then performed for all variants that are not judged to be heterogeneous. A potential problem with this sequential (that is, one-by-one) removal strategy is that, when there are several variants with similar outlying estimates, no single variant may be judged to be an outlier on its own. Alternative methods have considered penalized regression for simultaneous parameter estimation and outlier detection, using a Lasso (also called L _1_) penalty ^22^ or an L _0_ penalty. The constrained maximum likelihood (MR-cML) ^25^ is such a method that performs selection of invalid instruments and estimation allowing any of the three instrument variable assumptions to be violated via either uncorrelated or correlated pleiotropy. In addition to asymptotically valid inference, it also offers a data perturbation/resampling scheme to account for uncertainty in model selection and so achieve better inferences in finite samples.

A further class of robust methods uses latent modelling to distinguish to what extent genetic associations with the outcome arise due to a causal effect of the exposure, as opposed to pleiotropic effects of particular variants either on the outcome directly or on a common cause of the exposure and outcome. A causal model is evidenced if the predominance of variants that associate with the exposure also associate with the outcome in a proportional way. If the genetic associations with the outcome do not follow this pattern, then a non-causal explanation would be preferred. Emerging methods that take this approach include the Causal Analyses Using Summary Effect Estimates (CAUSE) ^88^ and Latent Heritable Confounder Mendelian randomization (LHC-MR) ^93^ methods.

While it would be excessive to perform every robust method for Mendelian randomization that has been proposed, or even all the methods mentioned here, investigators should pick a sensible range of methods to assess the sensitivity of their findings. For example, one suggestion is to perform MR-RAPS, the weighted median-based method, and the MR-cML method, as these methods require different assumptions to be satisfied for asymptotically consistent estimates (respectively: InSIDE, majority valid, and plurality valid). If estimates from all methods are similar, then any causal claim is more credible. However, finding differences between estimates does not necessarily imply the absence of a causal effect. Different methods will perform better and worse in different scenarios, so critical thought and judgement is required. Two recent simulation studies that compared different methods recommended the contamination mixture method ^26^ and MR-Mix ^94^ as having the lowest mean squared error across a range of different methods – these methods both make the same assumption for consistent estimation as the mode-based method, and so either could be used in preference to it. Alternatively, the MR-CUE ^95^ (“correlated horizontal pleiotropy unraveling shared etiology and confounding”) method has been demonstrated to have good performance in an extensive comparison of methods.

New methods for Mendelian randomization analysis are appearing regularly, and (unsurprisingly) tend to report simulations and applications suggesting they have advantages over previous methods. It is unlikely that one approach will perform best in all settings, and so it is important to perform a range of analyses that depend on different sets of assumptions. Combining this with orthogonal validation – such as through the use of positive and negative controls ( Section 7) – is the most powerful way of addressing causal questions, within the triangulation of evidence framework ^96, 97^.

We also recommend that a measure of the heterogeneity between variant-specific causal estimates, such as Cochran’s Q statistic or the I ^2^ statistic, is reported as a part of a polygenic Mendelian randomization investigation ^79, 98, 99^. Conclusions are more reliable when multiple genetic variants provide concordant evidence for a causal effect, and particularly when there is no more heterogeneity between the variant-specific causal estimates than expected by chance. As discussed in Section 5, some heterogeneity may be expected even when all genetic variants are valid instruments. However, causal conclusions are less reliable when there is substantial heterogeneity, especially when there are distinct outliers (which may represent pleiotropic variants) or when evidence for a causal effect depends on one or a small number of variants.

Leave-one-out analyses (i.e. remove one variant from the analysis and re-estimate the causal effect) can be valuable in assessing the reliance of a Mendelian randomization analysis on a particular variant ^100^. If there is one genetic variant that is particularly strongly associated with the exposure, then it may dominate the estimate of the causal effect. Investigators should assess the robustness of findings to the removal of such variants. If a causal effect is only evidenced by one variant, then the validity of the inference depends only on that variant. If there are many variants in an analysis, leaving one variant out at a time is unlikely to change the estimate substantially, and leaving out subsets of the variants (say, a randomly chosen 30% at a time ^101^) may be more appropriate. A further approach for identifying variants to remove from the analysis is Steiger filtering, which removes variants from the analysis if their association with the outcome is stronger than that with the exposure ^102^. It is highly unlikely that variants could have a stronger association with the outcome than the exposure if the instrumental variable assumptions are satisfied and the genetic association with the outcome is entirely mediated via the exposure (unless there is substantial measurement error in the exposure).

While removing horizontally pleiotropic variants from a Mendelian randomization analysis will improve the validity of causal inferences, there is some danger in a *post hoc* or data-driven selection of genetic variants. This is particularly true if many genetic variants are judged to be heterogeneous: the removal of too many variants from the analysis could provide a false impression of agreement amongst the remaining variants, and over-precision in the causal estimate. Removing a variant from the analysis is more justified when a pleiotropic association of the variant has been identified ^103^.

## 7. Other approaches for sensitivity analysis

Sensitivity analysis should not be limited to the application of different statistical methods. This is particularly important for investigations based on a single gene region, as several of the methods discussed above are not applicable in this case. Other approaches for assessing robustness include varying the dataset and choice of genetic variants in the analysis (including the suggestion of liberal and conservative variant sets in Section 3), the use of positive and negative control outcomes and/or samples, colocalization, subgroup analyses, and examining associations with potentially pleiotropic variables. We describe each of these in turn.

A positive control outcome is an outcome for which it is already established that the exposure is causal. For example, the outcome of gout may be used as a positive control in a Mendelian randomization investigation for serum uric acid as an exposure, as raised uric acid levels are known to increase risk of gout. Provided that there is sufficient statistical power, then if genetic variants that are associated with serum uric acid are not also associated with risk of gout, then we may question whether the genetic variants are truly able to assess the effects of varying serum uric acid ^104^. Conversely, a negative control outcome is an outcome for which it is believed that the exposure cannot be causal ^105^. For example, childhood levels of vitamin D have been used as a negative control outcome for the effect of adulthood BMI; childhood BMI was shown to affect childhood vitamin D levels in a Mendelian randomization investigation, but adulthood BMI did not ^106^. If a Mendelian randomization investigation suggests that the negative control outcome is caused by the exposure, then violation of the instrumental variable assumptions (such as through pleiotropy or population stratification) may be suspected ^107^.

Colocalization assesses whether the same genetic variant (or variants) influences two traits ^108, 109^. If genetic variants in a given gene region are associated with both an exposure and an outcome, it may be that the same genetic variants causally influence both the exposure and outcome (implying the likely presence of a causal pathway including the exposure and outcome). However, it may instead be that the two associations are driven by different causal variants, and these variants are correlated due to linkage disequilibrium ^110^. This would typically indicate a violation of the Mendelian randomization assumptions. An example of this is the *APOE* gene region, which contains genetic variants associated with LDL-cholesterol and Alzheimer’s disease. However, LDL-cholesterol does not appear to be a cause of Alzheimer’s disease in Mendelian randomization analyses using variants from other gene regions ^111^. A colocalization analysis revealed distinct causal variants for LDL-cholesterol and Alzheimer’s disease at the *APOE* locus, indicating that variants in this gene region are not valid instruments assessing the effect of LDL-cholesterol on Alzheimer’s disease ^112^.

Colocalization can be a useful sensitivity analysis when a Mendelian randomization analysis is based on a single gene region ^113^. However, there are several limitations to such an analysis, including identifying the true causal exposure (this is particularly relevant when using gene expression as the exposure, as colocalization results often differ depending on the choice of tissue) and statistical power. Bayesian approaches for colocalization (such as the *coloc* family of methods) typically only conclude that there is colocalization if there are variants having strong associations (p < 10 ^-4^ or 10 ^-5^) with both the exposure and outcome. In the language of *coloc*, if genetic variants are strongly associated with the exposure but not the outcome, then the method may prioritize hypothesis H _1_ (existence of a causal variant for trait 1 but not trait 2) rather than hypothesis H _3_ (distinct causal variants for traits 1 and 2 – that is, the traits fail to colocalize) or hypothesis H _4_ (shared causal variants for traits 1 and 2 – that is, the traits colocalize). Hence, while in some cases colocalization methods will provide helpful evidence supporting or questioning the Mendelian randomization assumptions, in other cases they may provide no strong evidence for or against colocalization ^112^.

A subgroup analysis comparing Mendelian randomization results from subgroups of the population in which the genetic variants have different degrees of association with the exposure can serve as a sensitivity analysis to assess the instrumental variable assumptions. An example of such a subgroup analysis is the comparison of genetic associations with blood pressure in men and women in an East Asian population for variants implicated in the metabolism of alcohol ^114, 115^. As women in East Asia tend not to drink alcohol, genetic associations with blood pressure are observed in men but not in women. Also, genetic associations are stronger in heavier drinkers ^114^. This provides confidence that the genetic associations are driven by alcohol consumption and not by a pleiotropic mechanism. Such an analysis can be performed if there is a subgroup of the population that has reduced or increased levels of the exposure ^116, 117^. However, if the subgroup is defined by a collider (see below), then stratification can introduce bias to the analysis ^118^. As sex cannot be affected by autosomal genetic variants, sex cannot be a collider, and so stratification on sex will not induce collider bias ^119^.

A further possible sensitivity analysis is to check the genetic associations with other variables associated with the outcome, and which are thought not to lie on the causal pathway through the exposure (i.e. are not mediators). Such variables may lie on alternative pleiotropic pathways to the outcome. If the genetic variants are not associated with such variables, then some reassurance can be drawn that the Mendelian randomization assumptions are satisfied. A further possibility in this case is to perform a multivariable Mendelian randomization, including the putative pleiotropic variables as additional exposures in the analysis model ^120^. This analysis will estimate the direct effect of the exposure on the outcome keeping these variables constant.

There are several other potential sources of bias in a Mendelian randomization analysis other than invalid instruments. We consider here collider bias, selection bias, and reverse causation as three potential sources of bias, and direct readers to reviews that list further potential sources of bias ^68, 121^.

A collider is a common effect of two variables – for example, the exposure is influenced by the genetic variants and the exposure—outcome confounders, and so is a collider. Any variable causally downstream of the exposure is also affected by the genetic variants and confounders, and so is also a collider. Even if the genetic variants and confounders are uncorrelated (they are marginally independent in the population), they will typically be associated when conditioning on the collider (they become conditionally dependent) ^118^. Stratifying on or adjusting for a collider therefore leads to an association between variables that influence the collider. An association between the genetic variants and the exposure—outcome confounders would lead to biased causal estimates ^122^. Collider bias is not unique to Mendelian randomization, but it is particularly relevant as some published genetic association estimates have been adjusted for potential colliders ^123^. For example, genome wide association studies of many brain volume measures routinely adjust for measures of cranial size or total brain volume, but head size may itself be influenced by exposures of interest in subsequent Mendelian randomization studies ^124^. Methods to account for collider bias have recently been proposed ^125– 127^.

Selection bias is a specific example of collider bias which occurs when selection into a study sample depends on a collider. Most epidemiological studies do not recruit all individuals from the target population with equal probability, and so suffer from selection bias. Even if genetic variants behave as if randomly distributed in the population as a whole, they may not be randomly distributed in a selected subset of the population. A specific example of selection bias is index event bias, where entry into the study sample is dependent on having a particular index event ^128^. For example, investigations into disease survival can only include individuals who have had an initial disease event ^129^. Simulation studies have shown that selection bias can have a severe impact on Mendelian randomization estimates, but only when the selection effects are quite strong ^72, 122^. Selection bias can potentially be addressed using inverse-probability weighting ^130^, although this requires estimation of the probability of selection into the study sample for all participants. Specific weights to reduce selection bias have been proposed for the UK Biobank study ^131^.

While the genetic code is fixed at conception and so cannot be influenced by reverse causation, if the outcome influences the risk factor, then variants that primarily affect the outcome would (in a large enough sample size) be associated with the exposure ^132^. As discussed above and shown in Figure 4f, if genetic variants used as instrumental variables for the exposure in fact influence the outcome primarily, then genetic associations with the outcome could be present without the exposure influencing the outcome. The MR-Steiger method has been developed to detect such variants (for a continuous exposure and outcome) and remove them from the analysis ^102^.

## 8. Extensions and additional analyses

We distinguish between sensitivity and supplementary analyses discussed in the previous two sections, which are conducted to improve reliability in testing the primary causal hypothesis, and extensions and additional analyses, which address related, but distinct causal hypotheses. We only provide brief comments and references on these extensions to Mendelian randomization, several of which are the subject of ongoing methodological investigations.

Non-linear Mendelian randomization aims to characterize the shape of the causal relationship between the exposure and outcome; that is, does the causal effect of the exposure on the outcome vary at different levels of the exposure ^39^? Several methods for non-linear instrumental variable analysis have been proposed. Two broad categories of non-linear methods operate by: i) estimation of a flexible model relating the exposure to the outcome ^133, 134^, and ii) stratification of the population into strata with different average levels of the exposure, and estimation of stratum-specific causal effects ^135^. Results from these approaches can be sensitive to the parametric assumptions made by the methods – for the first category, which models relating the exposure to the outcome are considered ^136^; and for the second category, whether the genetic effect on the exposure varies in the population ^137^. Indeed, variability in the effect of genetic variants on the exposure is evident for several exposures; this variability can lead to highly misleading estimates ^137, 138^. The doubly-ranked stratification method has been proposed that may be less sensitive to variability in these genetic effects ^139^. Assessment of the reliability of current non-linear methods is a topic of current research.

Factorial Mendelian randomization takes genetic predictors of two exposures (or two interventions on the same exposure), and assesses whether there is statistical interaction between these in their association with the outcome. Under the assumption that the genetic predictors are instrumental variables for the exposures, the statistical interaction can be interpreted as an interaction between the causal effects of the exposures on the outcome on the same scale ^140^. For example, a study investigated interactions between genetic variants in the *HMGCR* gene region and the *PCSK9* gene region, which respectively can be regarded as proxies for statins and PCSK9 inhibitors ^141^, in their associations with coronary artery disease risk. The investigation found no association of the outcome with the interaction term between these variants in logistic regression, indicating no evidence for deviation from additivity in the combined effects of statins and PCSK9 inhibitors on a logit scale. A weakness of these investigations is that statistical power to detect an interaction is often low, in which case a null finding should not be interpreted as strong evidence for lack of interaction.

Time-varying Mendelian randomization aims to assess the potentially varying effect of an exposure at different periods during the life course ^142^. For example, an investigation considered genetic predictors of BMI measured during early-life and later-life, and used a multivariable Mendelian randomization framework to assess the independent effects of early-life and later-life BMI on coronary artery disease risk ^143^. Little evidence was found for a direct effect of early-life BMI on coronary artery disease risk. In contrast, for breast cancer, the effect of early-life BMI appeared stronger than the effect of later-life BMI. Researchers should be cautious when performing such analyses to ensure that the values of the exposure at different time periods genuinely represent biologically distinct risk factors, and not simply measures taken at different times but capturing the same essential risk factor ^144^.

Mediation can be assessed in a Mendelian randomization framework in two ways ^145^. In two-step Mendelian randomization, analysts estimate the effect of the exposure on the outcome, and compare this to the product of the effect of the exposure on the proposed mediator multiplied by the effect of the mediator on the outcome ^146^. Each of these steps can be performed using standard Mendelian randomization, although separate instrumental variables are required for the exposure and mediator. These approaches can also be used to explore evidence for molecular mediation: the involvement of gene expression, DNA methylation or metabolite involvement in a disease pathway ^147^. Alternatively, analysts can compare the effect of the exposure on the outcome from standard (that is, univariable) Mendelian randomization to the effect from multivariable Mendelian randomization including the mediator as an additional exposure variable ^84^. The latter estimate represents the direct (that is, unmediated) effect of the exposure on the outcome. For example, investigators considered the effect of time in education on coronary heart disease in a multivariable Mendelian randomization additionally accounting for BMI, systolic blood pressure, and smoking behaviour ^148^. They showed that a substantial proportion of the effect of education on coronary heart disease risk was mediated via one or other of these traits.

In bidirectional Mendelian randomization, investigators perform separate Mendelian randomization analyses to assess the effect of the exposure on the outcome, and the effect of the outcome on the exposure ^149^. These analyses require separate instrument variables for the exposure and for the outcome ^150^. For example, investigators considered associations between genetic predictors of educational attainment and short-sightedness, and between genetic predictors of short-sightedness and educational attainment ^151^. They found associations in the former case, but not in the latter. This suggests that time spent in education affects one’s eyesight, rather than poor eyesight affecting an individual’s propensity to spend more time in education. Resolving the direction of causation between these two factors using Mendelian randomization answered a question first posed over 400 years ago ^152^. Although in this example, evidence for a causal effect was found in one direction but not the other, this is not always the case. For example, there is evidence from Mendelian randomization that higher BMI has a causal effect on increasing smoking prevalence ^153^, and that cigarette smoking causally reduces BMI ^154^. However, in other cases, bidirectional Mendelian randomization findings may reflect the presence of shared aetiological pathways, rather than true causal effects in both directions ^155^.

## 9. Data presentation

**Figure 5.**
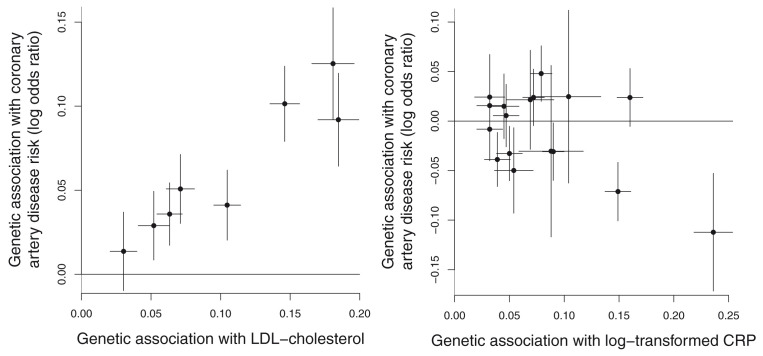
Scatter plot of genetic associations with the outcome (vertical axis) against genetic associations with the exposure (horizontal axis). Examples illustrated are: (left) no heterogeneity in the variant-specific causal estimates (effect of LDL-cholesterol on coronary heart disease risk using 8 variants associated with LDL-cholesterol); and (right) heterogeneity in the variant-specific causal estimates (effect of C-reactive protein on coronary heart disease risk using 17 genome-wide significant predictors of C-reactive protein). As indicated by differences in estimates, not all genetic variants are valid instrumental variables for C-reactive protein, and so a causal interpretation is not appropriate. Taken from Burgess *et al*., 2018 ^92^.

An attractive feature of Mendelian randomization is that the analysis can be summarized graphically in a transparent way. For example, in a polygenic analysis, a scatter plot of the genetic associations with the outcome against the genetic associations with the exposure reveals much about the analysis – whether different genetic variants provide similar estimates of the causal effect or if there is considerable heterogeneity, and whether the analysis is dominated by a single genetic variant or not ^31^. The scatter plot is appealing as it presents the data with no manipulation. Examples of scatter plots illustrating heterogeneity and no heterogeneity in the causal estimates from different variants are shown in Figure 5. Alternatives are forest plots, funnel plots, and radial plots – each of these assesses heterogeneity in the variant-specific causal estimates ^156^. Plots allow the investigators and readers to assess the reliability of the analysis method and its underlying assumptions, and we strongly recommend their inclusion in a manuscript.

Other important information to report include the first-stage R ^2^ statistic (when the exposure is continuous), which is a measure of the variance in the exposure explained by the genetic variants, and (particularly in a one-sample setting) the related F statistic, which is a measure of instrument strength and can be used to judge the extent of weak instrument bias ^157^. For multivariable Mendelian randomization, the conditional F statistic is a more relevant measure of instrument strength, and assesses the strength of association of the variants with each exposure in turn after accounting for the other exposures in the model ^158^.

Investigators can also make some statement about the power of their proposed analyses. Power to detect a causal effect depends on the proportion of variance in the exposure explained by the genetic variants, proposed size of causal effect, sample size (for the genetic associations with the outcome), and (with a binary outcome) proportion of individuals with an outcome event. Power calculators can be found at http://cnsgenomics.com/shiny/mRnd/ and https://sb452.shinyapps.io/power/. Power calculations are often performed *post hoc*, as sample sizes are rarely determined based on a proposed Mendelian randomization analysis. Power calculations are more meaningful when performed prior to the analysis, and can guide investigators which exposure/outcome pairs to consider, and so focus on analyses that have a better chance of giving meaningful results.

## 10. Interpretation

Finally, we discuss the interpretation of findings from Mendelian randomization investigations. In the first instance, a Mendelian randomization investigation assesses the association of genetic predictors of an exposure with an outcome, or equivalently, the association of genetically-predicted levels of an exposure with an outcome. Making causal inferences from observational data always relies on untestable assumptions. In Mendelian randomization, a key assumption is that observed differences in the outcome associated with genetically-predicted levels of the exposure would also be seen if the exposure were intervened on ^9, 71^. This version of the consistency assumption in causal inference ^159^ is referred to as gene—environment equivalence ^160^.

In line with the STROBE-MR guidelines ^1, 2^, we recommend that a cautious interpretation should be taken when describing the extent to which a causal effect has been demonstrated by a Mendelian randomization investigation. The appropriate degree of caution will depend on the plausibility of the instrumental variable assumptions, the concordance of estimates from different methods and different analytical approaches, the results from sensitivity and supplementary analyses, and so on. Even if a Mendelian randomization finding is replicated in a separate dataset, there is still intrinsic uncertainty in the instrumental variable assumptions, meaning that uncertainty in a causal conclusion remains. Another specific caution is that if multiple related traits are similarly associated with the same genetic variants (such as different measures of obesity or gene expression in different tissues), then Mendelian randomization approaches cannot identify the true causal risk factor without additional assumptions.

Mendelian randomization estimates relate specifically to changes in the exposure induced by the genetic variants used as instrumental variables. The genetic code is fixed at conception, and so Mendelian randomization investigations typically compare groups of the population having different trajectories in their distribution of the exposure over time ^161^. Analyses therefore typically can be interpreted as assessing the impact of long-term elevated levels of an exposure ^162^. For example, genetic variants in the *CRP* gene have been shown to be associated with CRP levels throughout the life course, with similar relative associations in childhood and in middle age ^163^. However, in most cases, we have incomplete information about how the genetic variant changes the distribution of the exposure across the life course. If the genetic associations with the exposure vary over time, then Mendelian randomization estimates based on genetic associations with the exposure measured at a single timepoint can be unreliable ^33^. Similar difficulties of interpretation arise if the impact on the outcome relates to levels of the exposure at a specific time period in life. A plausible example of this is the effect of vitamin D on multiple sclerosis; multiple sclerosis risk is hypothesized to be influenced by vitamin D levels during childhood, but not vitamin D levels in adulthood ^164^.

That said, results from Mendelian randomization investigations have often been shown to qualitatively agree with the results from randomized trials, suggesting that a causal interpretation for Mendelian randomization findings is often reasonable ^121^. Mendelian randomization investigations are worthwhile in providing an alternative line of aetiological evidence even though the instrumental variable assumptions can never be proved beyond all doubt ^96, 97^. However, quantitative differences between estimates from Mendelian randomization and from trials are likely, particularly as there are differences between how genetic variants influence the exposure and how clinical and pharmaceutical interventions influence the exposure ^165^ As genetic variants typically affect usual levels of exposures on a long-term basis, Mendelian randomization estimates are often larger than those from conventional observational studies or randomized trials for the same magnitude of difference in the exposure ^33^. Hence, the causal estimate from a Mendelian randomization investigation should not generally be interpreted directly as the expected impact of intervening on the exposure in applied practice ^166^.

The estimate from a Mendelian randomization investigation is therefore better interpreted as a test statistic for a causal hypothesis and an indicator of the direction of the effect, rather than the estimated impact of a well-defined intervention at a specific point in time. But even when a Mendelian randomization investigation is performed primarily to assess the causal role of an exposure, causal estimates can still be useful, for example to assess heterogeneity in estimates from different variants as a test of instrument validity, or to compare results from different analysis methods as an assessment of robustness. A logical consequence of the 2SLS/IVW method providing the most efficient causal estimate when combining evidence across multiple valid instrumental variables is that, under the same assumptions, the method provides the most powerful test of the presence of a causal effect.

## Summary

Overall, the key elements of a Mendelian randomization investigation to be reported in any manuscript are: i) motivation for why a Mendelian randomization analysis should be performed and for the scope of the analysis, ii) a clear description and justification of the choice of dataset(s) for the analysis, including why a one- or two-sample approach was chosen for the primary analysis, iii) a clear description and justification of the choice of genetic variants used in the analysis, iv) a discussion, whether statistically or biologically led, of whether the genetic variants are likely to satisfy the instrumental variable assumptions, v) a graphical presentation of the data, such as a scatter plot of the genetic associations, and vi) some attempt to test the robustness of the main findings, whether by use of robust methods (for a polygenic analysis) or another approach – whatever is most appropriate to the analysis under consideration. These elements are necessary for the reader to judge the reliability of a Mendelian randomization investigation.

Particularly with the advent of summarized data and the two-sample setting, performing a Mendelian randomization analysis has become more straightforward ^30^. The difficulty is not in performing a Mendelian randomization analysis, but rather in performing a credible analysis ^167^ and providing a reasoned interpretation ^164^. We hope that these guidelines, summarized in the accompanying flowcharts ( Figure 1 and Figure 2) and checklist ( Figure 3), will aid practitioners in performing reliable analyses, and editors and reviewers in judging the reliability of analyses, and that their use will help improve the overall quality of Mendelian randomization investigations.

## Disclaimer

The views expressed in this article are those of the authors. Publication in Wellcome Open Research does not imply endorsement by Wellcome.

## Data Availability

No data are associated with this article.
