# Supplementary material for: Guidelines for performing Mendelian randomization investigations: update for summer 2023
Source: Wellcome Open Res. 2023 Aug 4;4:186. Originally published 2019 Nov 26. [Version 3] doi: 10.12688/wellcomeopenres.15555.3 (PMC7384151; doi:10.12688/wellcomeopenres.15555.3)
Supplement: Peer review history

# Peer review: Reviewer response for version 2

**DOI:** 10.21956/wellcomeopenres.17423.r38569

**Published:** 2020-07-24

**Authors:** Sonja A. Swanson

**Affiliations:** 1 Department of Epidemiology, Erasmus University Medical Center, Rotterdam, The Netherlands

I appreciate the authors' willingness to engage with my suggestions in the prior review. I look forward to seeing this living document continue to improve as the methods, settings, and therefore guidelines relevant to Mendelian randomization investigators continue to evolve.

Is the rationale for developing the new method (or application) clearly explained?

Yes

Is the description of the method technically sound?

Partly

Are the conclusions about the method and its performance adequately supported by the findings presented in the article?

Partly

If any results are presented, are all the source data underlying the results available to ensure full reproducibility?

No source data required

Are sufficient details provided to allow replication of the method development and its use by others?

Partly

Reviewer Expertise:

Epidemiology; causal inference.

I confirm that I have read this submission and believe that I have an appropriate level of expertise to confirm that it is of an acceptable scientific standard.

# Peer review: Reviewer response for version 1

**DOI:** 10.21956/wellcomeopenres.17034.r37985

**Published:** 2020-03-17

**Authors:** Sonja A. Swanson

**Affiliations:** 1 Department of Epidemiology, Erasmus University Medical Center, Rotterdam, The Netherlands

These guidelines are a welcomed addition to the literature and reflect the experience and common practices performed by some thoughtful and influential researchers in the MR community. I am especially encouraged to see that this is a living document that the researchers expect to update periodically. Generally, the guidelines reflect consensus on analytic practices with some appropriate caveats provided for the targeted audience. In this review, I highlight areas that are not discussed in great detail that I think warrant attention in the development of further guidelines.

 1. **Well-defined causal effect estimands and causal null hypotheses**: Part 1 carefully separates the goal of testing for a non-null effect and estimating a causal effect. Its consideration of testing, however, does not make clear what types of null hypotheses can really be assessed (see Swanson *et al*. 2018) ^1^, and the description on effect estimation only suggests qualitatively weighing whether the genetic variants act like the intervention of interest. More guidance could be provided on question framing, especially by giving more specifics on the types of causal questions MR is able to answer. See also Hernán & Robins (2006) ^2^ and Swanson *et al*. (2017) ^3^.

 2. **The importance of time:** Related to the framing of the research question is also how time is a part of this framing. MR estimates are often described as “lifetime effects” but the standard IV methods used in MR are developed for time-fixed exposures. (Note that this type of concern also applies to causal null hypothesis testing, as described in Swanson *et al*. 2018) ^1^. At very least, some qualitative guidance on how to interpret results in light of the time-varying nature of exposures and outcomes over the life-course is needed. See again Swanson *et al*. (2017) ^3^and also Labrecque & Swanson (2019) ^4^.

 3. **Available robust methods, sensitivity analyses, and falsification strategies:** These guidelines state that Sections 6 and 7 are not exhaustive considerations of these tools, but it would be helpful to the MR community if guidance on these points was expanded in future versions of these guidelines. It is not very clear why the authors chose to present the tools they did have room for, and not others. It also is not very clear why these sections focus so much on the risk of bias due to pleiotropy when other types of biases can also threaten MR estimates.

Is the rationale for developing the new method (or application) clearly explained?

Yes

Is the description of the method technically sound?

Partly

Are the conclusions about the method and its performance adequately supported by the findings presented in the article?

Partly

If any results are presented, are all the source data underlying the results available to ensure full reproducibility?

No source data required

Are sufficient details provided to allow replication of the method development and its use by others?

Partly

Reviewer Expertise:

Epidemiology; causal inference.

I confirm that I have read this submission and believe that I have an appropriate level of expertise to confirm that it is of an acceptable scientific standard, however I have significant reservations, as outlined above.

## References

[1] : Causal null hypotheses of sustained treatment strategies: What can be tested with an instrumental variable?. *Eur J Epidemiol* .2018;33(8) : 10.1007/s10654-018-0396-6 723-728 10.1007/s10654-018-0396-6 29721747PMC6061140

[2] : Instruments for causal inference: an epidemiologist's dream?. *Epidemiology* .2006;17(4) : 10.1097/01.ede.0000222409.00878.37 360-72 10.1097/01.ede.0000222409.00878.37 16755261

[3] : Nature as a Trialist?: Deconstructing the Analogy Between Mendelian Randomization and Randomized Trials. *Epidemiology* .28(5) : 10.1097/EDE.0000000000000699 653-659 10.1097/EDE.0000000000000699 28590373PMC5552969

[4] : Interpretation and Potential Biases of Mendelian Randomization Estimates With Time-Varying Exposures. *American Journal of Epidemiology* .2019;188(1) : 10.1093/aje/kwy204 231-238 10.1093/aje/kwy204 30239571

# Response

**Published:** 2020-04-20

**Authors:** Stephen Burgess

**Affiliations:** University of Cambridge Addenbrooke's Hospital Cambridge, UK

> We thank the reviewers for their comments, which we have numbered for reference. We provide point-by-point responses to their suggestions below. <

Reviewer 2 (Sonja Swanson)

B0. These guidelines are a welcomed addition to the literature and reflect the experience and common practices performed by some thoughtful and influential researchers in the MR community. I am especially encouraged to see that this is a living document that the researchers expect to update periodically. Generally, the guidelines reflect consensus on analytic practices with some appropriate caveats provided for the targeted audience. In this review, I highlight areas that are not discussed in great detail that I think warrant attention in the development of further guidelines.

> We thank the reviewer for her comments and positive view of these guidelines. <

B1.       Well-defined causal effect estimands and causal null hypotheses: Part 1 carefully separates the goal of testing for a non-null effect and estimating a causal effect. Its consideration of testing, however, does not make clear what types of null hypotheses can really be assessed (see Swanson et al. 2018)1, and the description on effect estimation only suggests qualitatively weighing whether the genetic variants act like the intervention of interest. More guidance could be provided on question framing, especially by giving more specifics on the types of causal questions MR is able to answer. See also Hernán & Robins (2006)2 and Swanson et al. (2017)3.

> We appreciate this point, which we now introduce at the beginning of the manuscript, referencing the Swanson 2018 paper. We have added to the manuscript: “The straightforward statement of the causal hypothesis is that interventions in the exposure variable will affect the outcome. If the genetic associations with the exposure vary with time, then there are some nuances in terms of what causal hypotheses can be tested [Swanson et al, 2018]; we discuss the impact of time-varying relationships between variables in Section 9.” (Section 1). <

> We also refer to this debate in Section 9 (Interpretation) when we discuss numerical estimates from Mendelian randomization and how these relate to clinically-meaningful causal parameters. In particular, we have added to the manuscript: “The estimate from a Mendelian randomization investigation is therefore better interpreted as a test statistic for a causal hypothesis rather than the estimate of a well-defined intervention.” (Section 9).

B2.       The importance of time: Related to the framing of the research question is also how time is a part of this framing. MR estimates are often described as “lifetime effects” but the standard IV methods used in MR are developed for time-fixed exposures. (Note that this type of concern also applies to causal null hypothesis testing, as described in Swanson et al. 2018)1. At very least, some qualitative guidance on how to interpret results in light of the time-varying nature of exposures and outcomes over the life-course is needed. See again Swanson et al. (2017)3and also Labrecque & Swanson (2019)4.

> As per the response to point B1, we now reference the discussion about time-varying relationships between variables in Section 1 in relation to tests of the causal null hypothesis.

> We have edited the discussion in Section 9 to include issues relating to time-varying relationships between variables, referencing the papers cited above: “Mendelian randomization estimates relate specifically to changes in the exposure induced by the genetic variants used as instrumental variables. Genetic variants are present from before birth, and so Mendelian randomization investigations typically compare groups of the population having different trajectories in their distribution of the exposure over time. Analyses therefore typically can be interpreted as assessing the impact of long-term elevated levels of an exposure. However, in most cases, we have incomplete information about how the genetic variant changes the distribution of the exposure across the life course. If the genetic associations with the exposure vary over time, then Mendelian randomization estimates based on genetic associations with the exposure measured at a single timepoint can be unreliable. Similar difficulties of interpretation arise if the impact on the outcome relates to levels of the exposure at a specific time period in life. A plausible example of this is the effect of vitamin D on multiple sclerosis; multiple sclerosis risk is hypothesized to be influenced by vitamin D levels during early childhood, but not vitamin D levels in adulthood.” (Section 9). <

B3.      Available robust methods, sensitivity analyses, and falsification strategies: These guidelines state that Sections 6 and 7 are not exhaustive considerations of these tools, but it would be helpful to the MR community if guidance on these points was expanded in future versions of these guidelines. It is not very clear why the authors chose to present the tools they did have room for, and not others. It also is not very clear why these sections focus so much on the risk of bias due to pleiotropy when other types of biases can also threaten MR estimates.

> As we have stated, we will revise these guidelines over time. However, balance is needed in this manuscript between comprehensiveness and comprehensibility. It is perhaps better for a comprehensive discussion of methods to be provided separately. Also, while we aim to update these guidelines regularly, we are unable to update advice with the regularity needed to offer up-to-the-minute recommendations on methods as they are developed and updated. We therefore focus on methods whose performance is generally understood. We have now added reference to a more extensive review of methods: “This table is based on a broader review and comparison of methods [Slob, 2020].” (Section 6).

> We have now more clearly stated the motivation behind our choice of presentation: “We focus on these methods here as they can be implemented using summarized data alone, and they rely on different assumptions to provide consistent causal estimates.” (Section 6).

> In terms of focusing on pleiotropy, we were unclear in the initial submission. As described in Kang et al, JASA 2016 “Instrumental variables estimation with some invalid instruments and its application to Mendelian randomization”, there is a statistical correspondence between pleiotropy and instrument validity, meaning that any instrument invalidity can be expressed algebraically in terms of pleiotropy. Hence, while we use the language of pleiotropy to provide mathematically precise statements of the assumptions needed for consistent estimates, this section does not only cover pleiotropy, but invalid instruments more generally.

> We have changed the section headings for Sections 6 and 7 to: “Robust methods for sensitivity analysis” and “Other approaches for sensitivity analysis” to make clear that these sections are not narrowly focused on pleiotropy, but cover other sources of instrument invalidity. We have also added the sentence: “Although robust methods typically use the term ‘pleiotropy’, there is a mathematical correspondence between instrument invalidity and pleiotropy [Kang et al, 2016], and so these methods can help assess sensitivity of findings to instrument invalidity more generally, and not simply invalidity that arises from horizontal pleiotropy. We here use the language of pleiotropy to make mathematically precise statements about the assumptions needed for methods to provide consistent estimates.” (Section 6).

> As per the response to point A1, we have added new paragraphs in Section 7 on alternative sources of bias. We hope this addresses the reviewer’s concerns. <

# Peer review: Reviewer response for version 1

**DOI:** 10.21956/wellcomeopenres.17034.r37168

**Published:** 2019-12-24

**Authors:** Jean Morrison

**Affiliations:** 1 Department of Human Genetics, University of Chicago, Chicago, IL, USA

This article is a practical review of Mendelian randomization (MR) and set of guidelines intended for practitioners. The writing is clear and well organized and fairly thorough. I think this is an invaluable resource to investigators who wish to carry out an MR analysis and provides a good survey of relevant literature. My comments are mostly minor, I feel this article is much needed contribution to the field.

 Major comments

- Reverse direction effects: To me it seemed to one major omission was discussion of reverse direction effects. These can easily cause false positives for methods like IVW and are especially an issue if one is using the "agnostic" variable selection method. I think it could be good to add a graph or two graphs to Figure 4 displaying a reverse effect (of outcome on exposure) and possibly also a feedback loop. This should be accompanied by a discussion of when reverse effects are something the investigator should think about and when they aren't. The sensitivity testing section should include a discussion of testing in the opposite direction if agnostic variable selection is used and if the pair of traits warrants that consideration. It would also be good to include a discussion of how to interpret a positive result in both directions.
- Collider bias when using summary statistics: When discussing individual level data approaches the authors give a set of recommended covariates and note that collider bias is a concern. A brief explanation of what a collider is and how it causes bias should be added. A parallel discussion should be added concerning summary statistic based analyses. In particular, it is important that investigators know which covariates were adjusted to compute the summary statistics and how to identify a potential collider. 

Minor comments:

- Slightly more attention should be given to analyses described early as "exploratory" in which the investigator scans through many potential causal effects and how these should be treated differently. Relatedly, more attention could be given to analyses that use agnostic variable selection method. These issues are linked because a phenome wide MR analysis is likely to use the agnostic approach. In my view, for studies like these, a robust method (or multiple robust methods) should always be used, the investigator should assume that some variants are pleiotropic.
- There is more danger in using a one sample approach with an agnostic variant set due to weak instrument bias, which should be mentioned. Methods exist that estimate and correct for this bias by using correlation among test statistics for variants that aren't associated with either trait. CAUSE (Morrison *et al. *bioRxiv 2019) ^1^ is one but there must be other approaches to this issue as well. 
- Both Egger regression and the modal estimator have much lower power than other methods. This is worth mentioning when discussing interpreting results from sensitivity analyses.
- It is worth mentioning in figure 4 that the "related variable" may not always be known. 
- In the paragraph mentioning the "three sample" approach, it would be interesting to include any results about how much bias in the causal effect might be created by selection bias if one simply selects significant variants from the exposure GWAS.

Is the rationale for developing the new method (or application) clearly explained?

Yes

Is the description of the method technically sound?

Yes

Are the conclusions about the method and its performance adequately supported by the findings presented in the article?

Yes

If any results are presented, are all the source data underlying the results available to ensure full reproducibility?

No source data required

Are sufficient details provided to allow replication of the method development and its use by others?

Yes

Reviewer Expertise:

statistical genetics, Mendelian randomization

I confirm that I have read this submission and believe that I have an appropriate level of expertise to confirm that it is of an acceptable scientific standard.

## References

[1] : Mendelian randomization accounting for horizontal and correlated pleiotropic effects using genome-wide summary statistics[preprint]. *Biorxiv* .2019; https://doi.org/10.1101/682237 10.1101/682237 Reference source

# Response

**Published:** 2019-12-24

**Authors:** Stephen Burgess

**Affiliations:** University of Cambridge Addenbrooke's Hospital Cambridge, UK

Jean,

Thanks for the rapid and thoughtful response. We will wait for other reviewers to comment, and then update the article to address your points in due course.

Best wishes, Stephen

# Response

**Published:** 2020-04-20

**Authors:** Stephen Burgess

**Affiliations:** University of Cambridge Addenbrooke's Hospital Cambridge, UK

> We thank the reviewers for their comments, which we have numbered for reference. We provide point-by-point responses to their suggestions below. <

Reviewer 1 (Jean Morrison):

A0. This article is a practical review of Mendelian randomization (MR) and set of guidelines intended for practitioners. The writing is clear and well organized and fairly thorough. I think this is an invaluable resource to investigators who wish to carry out an MR analysis and provides a good survey of relevant literature. My comments are mostly minor, I feel this article is much needed contribution to the field.

> We thank the reviewer for her comments and positive view of these guidelines. <

Major comments

A1. Reverse direction effects: To me it seemed to one major omission was discussion of reverse direction effects. These can easily cause false positives for methods like IVW and are especially an issue if one is using the "agnostic" variable selection method. I think it could be good to add a graph or two graphs to Figure 4 displaying a reverse effect (of outcome on exposure) and possibly also a feedback loop. This should be accompanied by a discussion of when reverse effects are something the investigator should think about and when they aren't. The sensitivity testing section should include a discussion of testing in the opposite direction if agnostic variable selection is used and if the pair of traits warrants that consideration. It would also be good to include a discussion of how to interpret a positive result in both directions.

> We have now added new paragraphs to Section 7 covering biases in estimation arising due to issues other than invalid instruments. These covers reverse causation (point A1) and collider bias (point A2).

> Relating to reverse causation, we have added to the manuscript: “While the genetic code is fixed at conception and so cannot be influenced by reverse causation, if the outcome influences the risk factor, this can result in gene—outcome associations becoming distorted and lead to misleading inferences. As shown in Figure 4e, if genetic variants that are supposed to be instrumental variables for the exposure in fact influence the outcome primarily, then genetic associations with the outcome could be present without the exposure influencing the outcome. The MR-Steiger method has been developed to detect such variants and remove them from the analysis. Bidirectional Mendelian randomization analyses have been proposed that use separate sets of instrumental variables for the exposure and outcome to assess the direction of causal effect.” (Section 7).

> We have added a reverse causation scenario and a feedback loop scenario to Figure 4 as requested: “Another possible scenario that would lead to instrument invalidity is if genetic variants influence the outcome primarily rather than the exposure (Figure 4e, see also discussion on reverse causation in Section 7).” (Section 3). <

A2. Collider bias when using summary statistics: When discussing individual level data approaches the authors give a set of recommended covariates and note that collider bias is a concern. A brief explanation of what a collider is and how it causes bias should be added. A parallel discussion should be added concerning summary statistic based analyses. In particular, it is important that investigators know which covariates were adjusted to compute the summary statistics and how to identify a potential collider.

> As per the response to point A1, we have expanded the discussion about collider bias and selection bias in new paragraphs in Section 7 on bias due to issues other than invalid instruments.

> We have added to the manuscript: “A collider is a common effect of two variables – for example, any variable causally downstream of the exposure is influenced by the genetic variants and the exposure—outcome confounders, and so is a collider. Even if two variables are unrelated (they are marginally independent), they will typically be related when conditioning on the collider (conditionally dependent). Stratifying on or adjusting for a collider therefore leads to an association between variables that influence the collider. An association between the genetic variants and the exposure—outcome confounders would lead to biased causal estimates. Collider bias is not unique to Mendelian randomization, but it is particularly relevant as some published genetic association estimates have been adjusted for potential colliders. Methods to account for collider bias have recently been proposed.

“Selection bias is a specific example of collider bias which occurs when selection into a study sample depends on a collider. Simulation studies have shown that selection bias can have a severe impact on Mendelian randomization estimates, but only when the associations of variables with the collider are quite strong. Selection bias can potentially be addressed using inverse-probability weighting, although this requires estimation of the probability of selection into the study sample for all individuals.” (Section 7).

> We also discuss the problem raised by the reviewer about pre-computed summarized data: “If published summarized association estimates have already been adjusted for a variable causally downstream of the exposure, collider bias (see Section 7) may be unavoidable.” (Section 2). <

Minor comments:

A3. Slightly more attention should be given to analyses described early as "exploratory" in which the investigator scans through many potential causal effects and how these should be treated differently. Relatedly, more attention could be given to analyses that use agnostic variable selection method. These issues are linked because a phenome wide MR analysis is likely to use the agnostic approach. In my view, for studies like these, a robust method (or multiple robust methods) should always be used, the investigator should assume that some variants are pleiotropic.

> In reference to phenome scan analyses, we have added: “Such analyses are generally regarded as exploratory or “hypothesis-generating”, and results are typically treated as provisional until replicated in an independent dataset.” (Section 1). In relation to analyses performed using an agnostic set of genetic variants, we have added: “If genetic variants are chosen in a way that is completely agnostic to the function of the variants, then researchers should be especially careful about the possibility of variants being pleiotropic.” (Section 3). <

A4. There is more danger in using a one sample approach with an agnostic variant set due to weak instrument bias, which should be mentioned. Methods exist that estimate and correct for this bias by using correlation among test statistics for variants that aren't associated with either trait. CAUSE (Morrison et al. bioRxiv 2019)1 is one but there must be other approaches to this issue as well.

> We have added discussion on winner’s curse and weak instrument bias when genetic variants are selected in the dataset under analysis (see point A7).

> We have added reference to the CAUSE method and another similar paper: “A further class of robust methods uses latent modelling to distinguish to what extent genetic associations with the outcome arise due to a causal effect of the exposure, as opposed to via direct (pleiotropic) or confounder-driven effects of particular variants. A causal model is evidenced if the predominance of variants that associate with the exposure also associate with the outcome in a proportional way. If the genetic associations with the outcome do not follow this pattern, then a non-causal explanation would be preferred. Emerging methods that take this approach include the Causal Analyses Using Summary Effect Estimates (CAUSE) [Morrison et al] and Latent Heritable Confounder MR (LHC-MR) [Darrous et al] methods.” (Section 6).

> We have also added reference to recently developed methods that are based on multivariable Mendelian randomization: “Methods have been proposed based on a multivariable approach in the context of gene expression data, including the MR-link [van der Graaf et al] and transcriptome-wide summary statistics-based Mendelian Randomization (TWMR) [Porcu et al] methods.” (Section 7). <

A5. Both Egger regression and the modal estimator have much lower power than other methods. This is worth mentioning when discussing interpreting results from sensitivity analyses.

> We have now mentioned this with respect to the MR-Egger method: “Estimates from the MR-Egger method are particularly affected by outlying and influential datapoints, and are prone to be imprecise, particularly when the variant—exposure associations are all similar in magnitude. This can lead to the method having low power to detect a causal effect.” (Section 6), and also with respect to the mode-based method: “The mode-based method has been shown to have low precision in some simulated and real datasets.” (Section 6). <

A6. It is worth mentioning in figure 4 that the "related variable" may not always be known.

> We now mention this in the figure caption: “We note that the related variable may be known or unknown.”. <

A7. In the paragraph mentioning the "three sample" approach, it would be interesting to include any results about how much bias in the causal effect might be created by selection bias if one simply selects significant variants from the exposure GWAS.

> Bias due to selecting variants based on their statistical significance in the dataset under analysis comes under the broader category of winner’s curse. Selecting variants based on their association with the exposure in the dataset under analysis can lead to exacerbation of weak instrument bias. We have expanded the discussion on winner’s curse in Section 3: “Often, selection is based on the dataset in which genetic associations with the exposure are estimated. However, this leads to “winner’s curse” –genetic associations tend to be overestimated in the dataset in which they were first discovered. If genetic variants are selected based on their associations with the exposure in the dataset under analysis, weak instrument bias is exacerbated (in the direction of the observational association in a one-sample setting, and in the direction of the null in a two-sample setting).” (Section 3). <
